# Using Ethical Reasoning to Amplify the Reach and Resonance of Professional Codes of Conduct in Training Big Data Scientists

**DOI:** 10.1007/s11948-014-9613-1

**Published:** 2014-11-28

**Authors:** Rochelle E. Tractenberg, Andrew J. Russell, Gregory J. Morgan, Kevin T. FitzGerald, Jeff Collmann, Lee Vinsel, Michael Steinmann, Lisa M. Dolling

**Affiliations:** 1 Collaborative for Research on Outcomes and -Metrics, Departments of Neurology, Biostatistics, Bioinformatics and Biomathematics, and Psychiatry, Georgetown University Medical Center, Building D, Suite 207, 4000 Reservoir Rd. NW, Washington, DC 20057 USA; 2 Dr. David P. Lauler Chair in Catholic Health Care Ethics and Department of Oncology, Georgetown University Medical Center, Washington, DC USA; 3 Department of Molecular Biology, Georgetown University Medical Center, Washington, DC USA; 4 College of Arts and Letters, Stevens Institute of Technology, Hoboken, NJ USA

**Keywords:** Ethics education, Big Data, Professionalism, Training, Curriculum

## Abstract

The use of Big Data—however the term is defined—involves a wide array of issues and stakeholders, thereby increasing numbers of complex decisions around issues including data acquisition, use, and sharing. Big Data is becoming a significant component of practice in an ever-increasing range of disciplines; however, since it is not a coherent “discipline” itself, specific codes of conduct for Big Data users and researchers do not exist. While many institutions have created, or will create, training opportunities (e.g., degree programs, workshops) to prepare people to work in and around Big Data, insufficient time, space, and thought have been dedicated to training these people to engage with the ethical, legal, and social issues in this new domain. Since Big Data practitioners come from, and work in, diverse contexts, neither a relevant professional code of conduct nor specific formal ethics training are likely to be readily available. This normative paper describes an approach to conceptualizing ethical reasoning and integrating it into training for Big Data use and research. Our approach is based on a published framework that emphasizes ethical reasoning rather than topical knowledge. We describe the formation of professional community norms from two key disciplines that contribute to the emergent field of Big Data: computer science and statistics. Historical analogies from these professions suggest strategies for introducing trainees and orienting practitioners both to ethical reasoning and to a code of professional conduct itself. We include two semester course syllabi to strengthen our thesis that codes of conduct (including and beyond those we describe) can be harnessed to support the development of ethical reasoning in, and a sense of professional identity among, Big Data practitioners.

## Introduction

In their 2012 article, “Critical questions for Big Data: Provocations for a cultural, technological, and scholarly phenomenon”, scholars at Microsoft Research and members of the Microsoft Social Media Collective reject the attempt to define “big data” as a phenomenon characterized by the size or complexity of the data, per se (Boyd and Crawford 2012). They note that long before the current era, massive data sets, such as the US Census, have been routinely collected and analyzed. Instead they define Big Data as a socio-technological phenomenon that integrates—or has the potential to integrate—technology, analysis, and scholarship. Boyd and Crawford (2012) emphasize that Big Data (capitalized to convey its status as a phenomenon to be studied, p. 675, footnote 1) involves scholars from social, quantitative, and technological domains—as well as participants from financial and business domains with limited interest in scholarship, publishing, or academic applications of Big Data. This diversity presents a significant problem: no single discipline exists to govern, or even to create norms around ethical practices in, Big Data use or research.

This normative paper describes an approach to using ethical reasoning to promote professionalism, and to prepare practitioners for responsible and ethical Big Data use, research, and engagement. We draw on the more scholarly domains where Big Data research and tools are developed and employed (e.g., Dourish and Bell 2011, Ch. 1) in order to anchor our approach; however, we believe this paper is also relevant for the more commercial and less academic facets of Big Data, because all *should* involve properly trained practitioners (e.g., Bollier 2010). We aim to orient readers to the role and relevance of ethical reasoning in a twofold way, both historically and methodologically. We show, first, the centrality of both codes of conduct and ethics in the process of professionalization and discipline formation, and will then outline a way to use existing codes of conduct for the ethical training of practitioners. We focus the discussion of the formation of professional community norms on two key disciplines from which Big Data may attract many practitioners, computer science and statistics (i.e., technology and analysis). However, the approach can be used to amplify the reach and resonance of the code of conduct from any field.

The development of a discipline or profession tends to proceed in tandem with a normative agreement on a code of professional ethics or conduct for the practitioner in the community (Starr 1984; Parker 1968; Abbott 1988; see also Berg and Singer 1995; Krimsky 1984; National Society of Genetic Counselors 1992), but with Big Data attracting practitioners from many diverse backgrounds, the development of a single discipline or profession specific code of conduct or ethics appears unlikely. As a result, norms around ethical practices in Big Data use or research are likely to develop in a fragmented or piecemeal manner, if they develop at all (see, e.g., Lo 1993). In that case, institutional practice and culture would be the only exposure that Big Data practitioners have for developing community and normative behaviors like “professionalism,” or professional conduct.

For undergraduate and graduate students, particularly those in the sciences, the dominant training model for “ethics” in scholarly work, the *responsible conduct of research,* is a single static training opportunity (e.g., course or module), often for all “researchers” or science students within an institution. However, not all scientific fields involve research, so if an institutional training program for ethics is characterized as supporting only “research”, it limits the relevance—whether perceived or actual—of this training. This limitation may be particularly true for those involved in, or preparing to engage with, Big Data—whether it is viewed as a new paradigm, a new application, or a new “cultural, technological and scholarly phenomenon” (Boyd and Crawford 2012).

Lo (1993) outlines (and addresses) many objections to the formal integration of ethics training into preparation for scientific research (and by extension, the practice of science). By integrating a published model for training in *ethical reasoning* with codes of professional conduct with which Big Data practitioners and instructors might be familiar, this paper describes a way forward. Ethical reasoning is a learnable and improvable skill set (Tractenberg and FitzGerald 2012) that can be sustainable (Tractenberg et al. in review) beyond the course, and so can serve to both introduce future practitioners to codes of professional conduct, and also prepare them for challenges in their practice that cannot be foreseen.

The dominant training model for “ethics” in scholarly work, the *responsible conduct of research,* usually requires a single training opportunity, often in the context of early preparation for, or gaining approval to conduct, research. Because major applications of Big Data exist outside of formal “research”, a research-oriented institutional training program for ethics may miss important issues and substantial practitioner communities that do/will engage with Big Data. The common model of “institutional training in the responsible conduct of research” typically only weakly relates to practitioners with Big Data and quantitative scientists. Professional associations that specify codes of ethical conduct may relate just as weakly because they are discipline specific. By contrast, the fact that Big Data is becoming *transdisciplinary* or *discipline independent* suggests a general, rather than a domain-, discipline-, or profession-specific model of training and practice in ethical reasoning would be more effective. The ethical reasoning approach we describe targets the decision-making that is inherent to the practitioner’s work (Bollier 2010; see also Boyd and Crawford 2012; Dwork and Mulligan 2013), so that this training can be applied across a wide range of contexts, even if these were not part of the original training program.

The Association of Internet Researchers (AoIR) directly challenges, and offers an alternative to, the dominant responsible conduct of research training model. Specifically, the AoIR’s revised code of conduct, “Ethical Decisionmaking and Internet Research” (AoIR Ethics Committee 2012), includes a set of recommendations focused on the ongoing and iterative decision-making that is required *throughout* research that involves the Internet (or data derived therefrom), with only passing mention of the core “factual” documentation that comprises the bulk of most responsible conduct of research training “courses” or modules. Moreover, the AoIR specifically targets *ethical reflection* (contemplation of the ethical implications of decisions made throughout any given research project), and not factual mastery of any sort (which is a more standard approach to the ‘topics to be covered’ in a course on the responsible conduct of research). The National Academy of Engineering (NAE) (NAE 2013) questioned the goals and utility of the typical training course. “…(t)he entire community of scientists and engineers benefits from diverse, ongoing options to engage in conversations about the ethical dimensions of research and (practice),” (Kalichman 2013: 13). The perspectives of both engineering and Internet research emphasize ethical reflection and building capacity for ongoing discussion.

From yet another disciplinary perspective, we, too, published an outline of learnable, improvable reasoning skills (Tractenberg and FitzGerald 2012) that can lead to, and support, the type of ethical reflection the AoIR describes and that the NAE (2013) recommends (see also King and Kitchener 1994). Our approach to ethical reasoning is based on a published career-spanning (developmental) paradigm for training in ethical reasoning: the Mastery Rubric (MR) for ethical reasoning (MR-ER, Tractenberg and FitzGerald 2012). A Mastery Rubric is a curriculum building and evaluation tool, similar to a traditional rubric (e.g., Stevens and Levi 2005) in that the desired knowledge, skills and abilities for acurriculum—rather than an assignment or task—are outlined together with performance levels that characterize the respondent from novice to proficiency (Tractenberg et al. 2010). The Mastery Rubric for Ethical Reasoning treats ethical reasoning (ER) as a learnable, improvable skill set: (identification and assessment of one’s) prerequisite knowledge; recognition of a moral issue; identification of relevant decision-making frameworks; identification and evaluation of alternative actions; making and justifying a decision (about the moral issue); reflection on the decision. These were derived from compendia of scholarly work reflecting ethical decisionmaking (http://www.scu.edu/ethics/). This skill set—if developed and fostered—can be utilized to support decision-making in and around Big Data, in contexts that range from career-specific issues of professionalism (e.g., AoIR 2012; Tractenberg 2013) to ethical, legal, and social issues that require discussion and input representing a wide range of disciplinary perspectives (e.g., “ongoing options to engage in conversations about the ethical dimensions of research and (practice)” (Kalichman 2013: 13)).

This approach is as applicable in other science domains (Tractenberg et al. in review) as it would be in Big Data, and was designed and intended to be utilized throughout the course of a scientific and professional career whether the domain is the Internet, biomedical research, or some combination of these domains with others. Given the importance of Big Data, many institutions have created, or will create, training opportunities (e.g., degree programs, workshops) to prepare people to work in and around the domain. The limited “professional practice” guidelines for Big Data may be one reason why insufficient time, space, and thought have been dedicated to how to train new practitioners to engage with the ethical, legal, and social issues in this new domain. Our paradigm can be used to fill this gap.

## Methods

We summarize the histories of the codes of professional conduct from two disciplines with clear involvement in Big Data (collection and analysis): computer science and statistics. Our capsule histories (Results) provide historical analogies between the past and the present that focus on three points: the instability of communities at the early phases of emergent professions and disciplines; individual efforts to catalyze professional communities around clear questions; and the centrality of codes of conduct and ethics in the process of professionalization and discipline formation. In our use of historical analogies from computer science and statistics, we draw upon canonical and collaborative studies published by practitioners in each field, as well as by historians of technology, diplomatic historians, and political scientists that provide guidance for the benefits and limits of historical analogies for policymakers (Mazlish 1965; Neustadt and May 1986). To demonstrate how ethical reasoning can support this training for faculty and students alike, we integrated the ER skill set with the professional codes of conduct from the Association of Computing Machinery (ACM) and the American Statistical Association (ASA). Both the American Statistical Association (ASA, http://www.amstat.org/committees/ethics/) and the Association for Computing Machinery (ACM, http://www.acm.org/about/code-of-ethics) have extensive and well-crafted codes of professional conduct. The codes are among the best-articulated and most relevant to professional involvement with Big Data, and each can be seen as having specific relevance to professional conduct around Big Data. Because Big Data is multidimensional and multi-disciplinary, choosing “the profession” whose code of conduct most relates to one’s Big Data engagement is an excellent starting point; integrating ethical reasoning knowledge, skills and abilities with ANY code of conduct will help sustain the objectives of “training in ethics” beyond the completion of this training (Tractenberg et al. in review). This approach may therefore be useful in stimulating reflection on novel ethical, legal, and social issues (ELSI) that arise or are encountered beyond the context of training.

Finally, we took the mapping of the codes of conduct (ASA, ACM) with the MR-ER and examined their alignment with the seven criteria for teaching goals of ethical training outlined in the NAE conference report (NAE 2013), for ethics training for science, technology, engineering and mathematics (STEM) disciplines, within which Big Data clearly falls.

The seven criteria for characterizing teaching goals of ethical training (NAE 2013; Kalichman 2013: 11) are:

1. (Each ethics teaching) goal should represent something important/relevant to the ethical or responsible conduct of research or practice.
2. Goal should identify and address some concrete deficiency.
3. Achievement of the goal should be independent of other (possibly related) goals.
4. Goal should be actually and observably amenable to an active intervention.
5. Achievement of the goal should be documented/documentable with either quantitative or qualitative <as appropriate> outcomes.
6. Achievement of the goal should result in a change that is detectible and meaningful.
7. Goal should be feasible.

## Results

We present the historical summaries of the two codes of conduct first, followed by the mapping of these codes with the MR-ER knowledge skills and abilities, and finally present the assessment of the consistency of these maps with the NAE ethics teaching goals criteria (Kalichman 2013).

### Historical Summary 1: Association of Computing Machinery

The Association of Computing Machinery (ACM), today a prestigious international society with over 100,000 members, began humbly and informally at a meeting at Columbia University in the fall of 1947. The meeting’s organizer was Edmund C. Berkeley, an insurance industry expert whose first encounter with the power and charm of computers came during World War II when the Navy assigned him to work at Harvard on Howard Aiken’s Mark I. After the war, Berkeley returned to work at the Prudential Insurance Company, where he explored how Prudential could integrate new machine calculating technologies (Akera 2007). At that time, neither computer technology nor the very meaning of the term “computer” were stable. Moreover, a diverse and disperse group of military and government officials, mathematicians, engineers, and equipment manufacturers were building and using things that subsequent generations would recognize as early computers. Before Berkeley organized the 1947 meeting at Columbia, there was no obvious reason that this group of thinkers, builders, and users would soon coalesce into a single professional community.

Nevertheless, Berkeley and hundreds of fellow computer enthusiasts gave life to the ACM, and the organization grew and flourished during the 1940s and 1950s. Throughout this period the ACM followed the familiar patterns of professionalization established by communities of technical experts in the late 19th century. Organizations such as the American Society for Mechanical Engineering (founded 1880) and the American Institute of Electrical Engineers (founded 1884) thrived because they were able to carve out distinctive occupational niches and propagate effective mechanisms that could facilitate the growth of an expert community of practitioners. Standards for membership and codes of ethics were key institutional expressions of their shared professional identity (McMahon 1984; Sinclair 1980; Abbott 1988).

ACM members—computer experts in industry, academia, and government—confronted conditions of persistent instability as they created boundaries between “computer science,” “computer engineering,” “software engineering,” and related fields (Shapiro 1997; Mahoney and Haigh 2011; Ensmenger 2012; Jesiek 2013). A fundamental problem in the 1950s and 1960s was that the supply of graduates from academic programs was not well aligned with the growing industrial and commercial demand for programming labor. Indeed, as historian Nathan Ensmenger observed, “there was little agreement within the computing community about who exactly qualified as an experienced, professional practitioner” (Ensmenger 2001).

To cultivate a greater sense of prestige and stability within their field, ACM members formed special interest groups in specific technical fields, as well as committees to make recommendations about social issues such as educational curricula and professional conduct. The latter effort began in 1966, when the ACM Council adopted “Guidelines for Professional Conduct in Information Processing.” Donn B. Parker, Chairman of the ACM Professional Standards and Practices Committee, boldly laid out their motivation in a 1968 article: “There are a number of serious ethical problems in the arts and sciences of information processing. […] It is difficult to discuss ethics in our field without considering professionalism […] [but] the diverse backgrounds of people in the field and the diverse applications of computers in other professions are significant problems in an effort to unify.” Parker continued, “Sooner or later some body or group is bound to do something drastic and bring nationwide attention and disgrace to our profession. We are sitting on the proverbial powder keg. […] The press is creating a fear of computers through their personification with such headlines as ‘Meet the Monster that Checks Your Taxes’” (Parker 1968).

The ACM thus initiated their foray into professional ethics as an urgent response to the fear that Parker articulated so vividly: computers—and computer “professionals”—were vulnerable to public mistrust due to misunderstanding, unscrupulous applications, or both. The ACM formalized its 1966 Guidelines into a Code of Professional Conduct, adopted in 1972. Subsequent revisions refined the 1972 Code until the ACM took a new direction with its Code of Ethics and Professional Conduct in 1992 (ACM 1992).

ACM leaders emphasized the pedagogical function of an “educationally oriented code” that would clarify the social responsibilities of the profession. The ACM reframed its 1992 Code as “a basis for ethical decision making in the conduct of professional work,” organized around 24 “imperatives”: “general moral imperatives” concerning honesty, fairness, social responsibility, intellectual property, privacy, and confidentiality; “specific professional responsibilities” concerning the quality of work and unauthorized access to computer systems; and “organizational leadership imperatives” concerning fair and conscientious management of people and resources; and commitments to comply with and uphold the Code. “The future of the computing profession,” the 1992 Code concluded, “depends on both technical and ethical excellence” (Anderson et al. 1993).

The ACM has had mixed success with its plea for technical and ethical excellence in the computing profession. In the twenty-first century, technical and ethical shortcomings became notoriously common throughout computer design, operation, and use (Luca 2014; Scheuerman 2014; Singleton 2014). By design the ACM’s Code of Ethics is voluntary and aspirational—enforceable only through social and professional pressure. The ACM has helped to create and promulgate accreditation criteria for university computer science programs to train students to understand their “professional, ethical, legal, security, and social issues and responsibilities.” But there is no standard curriculum to teach the nuances of ethical reasoning, and high-profile instances of criminal behavior and systematic violations of user privacy undermine the ACM’s aspiration to contribute to social and human well-being.

### Historical Summary 2: American Statistical Association

The origins of statistics as a discipline and field are difficult to pinpoint, mainly because there are two common meanings for the term. One definition of statistics, in the form of census (summary) type data was used by the Ancient Greeks, Chinese and Romans. The second meaning of the term “statistic” is as an estimator for a population (i.e., “true”) parameter, and arose after the invention of calculus in 1684 (Leibniz) and 1687 (Newton) which led to the abilities to realize the second meaning of the term.

Possibly the most limiting factor in the development of statistics as a formal discipline was the intractability of the mathematical computations required for anything beyond the most straightforward descriptive statistics for distributions. The advent of computers greatly facilitated calculations that had previously been done by hand and/or with reference to published tables of probabilities. Americans in the 1920s were among the first to apply statistics and computing together to analyze economic, financial, and agricultural issues (Grier 1995).

Mason et al. (1990) describe the creation of the American Statistical Association (ASA) in 1839 while the Royal Statistics Society (RSS) was established in the UK 1834 (Chartered as the “Royal Statistical Society” in 1887). The RSS was created as a special section within the British Association for the Advancement of Science, while the ASA evolved with a close affiliation to, but independent of, the US Federal government. The International Statistical Institute (ISI) was created in 1885.

The original objectives of the ASA were “to collect, preserve, and diffuse statistical information in the different departments of human knowledge,” and to “promote the science of Statistics…” Governments (federal, state, and local) were the main users of statistics in the US in the mid 1800 s, but there were important implications for economics, agriculture, and public health in those same census data. The ASA supported the creation of the US Census Bureau and Bureau of Labor Statistics, and professional statisticians, have contributed critically to agriculture, economics and other social sciences, clinical and biomedical research, and many other fields. However, professionals from other fields, particularly up until the mid twentieth century, have also contributed critically to statistics as a discipline.

In the early to mid-1900 s, interactions between economists, sociologists, and theoretical statisticians (on statistical applications) prompted changes in statistics education. Since their inceptions in the UK (RSS) and the US (ASA), these statistical societies and their members were committed to the promotion of the discipline and to its rigor. The ASA actively encouraged and promoted statistics within other disciplines, particularly in the early part of the twentieth century; this led to the establishment of new disciplines or subsets of disciplines wherein computing or statistics (or both) are featured. The interactivity of statistics and statisticians with other domains is represented concretely in the current (2014) ASA objectives, “…to foster statistics and its applications, to promote unity and effectiveness of effort among all concerned with statistical problems, and to increase the contribution of statistics to human welfare… (it) cooperates with other organizations in the advancement of statistics, stimulates research, promotes high professional standards and integrity in the application of statistics, fosters education in statistics, and, in general, makes statistics of service to society.”

The ASA section on “the training of statisticians” (which later became the section on Statistical Education) was established in 1944, only the second section to be created in the ASA. The International Federation for Information Processing was created in 1960 by UNESCO, and the International Association of Statistical Computing was established within the ISI in 1977, but a committee on Computational Statistics and Data Mining for Knowledge Discovery was only established by the ISI in 2012. Thus, the disciplinary and professional status of statistics has changed over time and the reliance on computers and computing must be considered a primary driver of this evolution.

Possibly reflecting its origins close to Federal statistics and data, the passages of the Freedom of Information and Privacy Acts in 1966 and 1974, respectively, raised issues for professional practice among federal statisticians, and an ASA Ad Hoc committee on Privacy and Confidentiality was appointed. The committee report was published in 1977 (ASA 1977), and later that year, an Ad Hoc “Committee on the Code of Conduct” was appointed. Meanwhile, the Belmont Report (“Ethical Principles and Guidelines for the Protection of Human Subjects of Research”, U.S. Department of Health, Education, and Welfare 1979) was published in 1979, bringing with it significant new initiatives for all participants in research involving human subjects in biomedical and behavioral research. The Belmont Report went far beyond privacy and confidentiality, and ushered in an era of training in “responsible conduct of research” that applied (or was applied) to any and all federally funded research—and researchers—in the U.S. (see, e.g., U.S. Department of Health and Human Services 1979; Bugliarello 1993 and National Academy of Engineering 2013).

The ASA Committee on the Code of Conduct became the “Committee on Professional Ethics” in 1982, and in 1983, this committee published the first ever “Ethical Guidelines for Statistical Practice” (ASA 1983), although “Principles of Professional Statistical Practice” was published in the Annals of Mathematical Statistics in 1965 (Deming 1965). This report can be seen to have integrated elements from the Belmont Report (introducing “basic ethical principles” and the idea that practice and research are two separable aspects of biomedical research) and the ASA Privacy and Confidentiality report from 1977. In 1985, the ISI published its “Declaration on Professional Ethics” (revised in 2010, ISI 2010), and the Encyclopedia of Statistical Sciences (first edition 1986) has included “Principles of Professional Statistical Practice” in each edition (e.g., Demming 2006). Also in 1986, the Committee on Professional Ethics was made a continuing committee (instead of Ad Hoc). The ASA Guidelines were revised in 1999 and are being revised again in 2014. The current domains on which the ASA guidelines focus are: professionalism; competence, judgment, diligence; responsibilities to funders, clients and employers assuring that statistical work is suitable; responsibilities in publications and testimony; responsibilities to research subjects; responsibilities to research team colleagues; responsibilities to other statisticians or statistical practitioners; responsibilities regarding allegations of misconduct; and responsibilities of employers.

The evolution of this code of conduct reflects two key elements of statistical practice and the ASA’s origins: namely, US Federal initiatives in 1966 and 1974 and the Belmont Report in 1979; and the influence those acts had on federally funded research and the training of new federally funded researchers—primarily in biomedical, clinical, and behavioral research domains. The laws represent impetus for statisticians practicing with Federal data (government statisticians), and had the potential to affect academic statisticians—who tend to be supported by federal funds, as most academic researchers in applied statistical domains are.

There have been no efforts to integrate professional conduct or the ASA Ethical Guidelines for Professional Practice into statistics curricula at any level, but standardization efforts for statistics curricula have gone on periodically. The ASA initiated a process to create Guidelines for Assessment and Instruction in Statistics Education for pre K-12th grade (ASA 2007) and undergraduates (ASA 2005), to bring statistical training (to be achieved within a single course) to undergraduate non-majors, and to early education curricula. Perhaps because the target audience is clearly not practitioners, neither of these includes any elements of “professional ethics” or a code of conduct. However, *none* of the training initiatives of the ASA mention professional ethics, either. The current (2014) undergraduate statistics (for majors) curriculum reform would be the first to mention the guidelines.

### Mapping Ethical Reasoning and Its development to Codes of Conduct

These historical summaries outline how and why Big Data scientists-in-training, whose background may be focused more on data analysis (ASA) or on computing (ACM, as examples), might very well come to their practice with no knowledge of a discipline’s codes of conduct: the codes are not usually formally taught in any curriculum. Furthermore, Big Data practitioners might also be unaware of any discipline-specific code of conduct because they do not identify themselves strictly with one or another profession or professional society. This approach by no means is limited to computing and statistics, since it is well known that Big Data draws practitioners from a wide range of disciplines. The approach is also not unique to training in Big Data; our recent experiences involve students from neuroscience, molecular biology, immunology, and clinical and translational research (Tractenberg et al. in review).

Two examples of syllabi, geared towards graduate level students, are provided in the Appendices 1 and 2. Appendix 1 presents a semester course syllabus intended to introduce students to the eight fundamental moral imperatives of the ACM code, and also give them opportunities to learn, practice, and get feedback on the individual ethical reasoning skills. These skills are generally supportive of academic activities (writing, speaking, training others), and the ACM’s “General Moral Imperatives” are also widely applicable. However, the syllabus could be modified to capture other issues or domains of interest or relevance to the community (or the time), creating at least a semester’s worth of “…options to engage in conversations about the ethical dimensions of research and (practice)” (Kalichman 2013: 13).

Appendix 2 presents descriptions of meeting objectives for a semester course syllabus intended to teach the ethical reasoning KSAs using the eight domains of the ASA code of conduct and also, the NIH “responsible conduct of research topics” list. We did not include the overview, prerequisite/co-requisite, required text, and other details that the full syllabus given in Appendix 1 contains for simplicity. These ancillary and structural materials work equally well for the course designed with the ACM and the ASA codes and would also work for courses that integrate other professional codes with ethical reasoning training as well.

Instructors of a course on ethics for professional practice (in many different domains and disciplines) can take the syllabi we have created and teach these semester courses, or they can modify them so as to capture *any other code of conduct*. Appendix 2 shows a course designed to ensure that any federally-funded students who must take this “responsible conduct of research” training will not only fulfill this requirement, but will also learn ethical reasoning skills and a code of conduct that (*unlike* the “responsible conduct of research” topics, Antes et al. 2010) could be useful to them in their professional lives.

Table 1 aligns the two syllabi with the seven NAE criteria for ethics education goals articulated earlier (Kalichman 2013).

**Table 1:** Alignment of ethics course goals criteria (NAE 2013) with syllabi mapping MR-ER to codes of conduct

NAE criterion	ACM code/semester course syllabus	ASA code/semester course syllabus
1. Goal should represent something important/relevant to the ethical or responsible conduct of research or practice	Ensures that at least one code of professional conduct will be introduced and discussed. Ensures that the main domains of the code of conduct will be developed up to a specific target level	
2. Goal should identify and address some concrete deficiency	There is no current (2014) integration of either code of conduct (of ACM or ASA) in any program of study; ethical reasoning is generally not taught to quantitative scientists	
3. Achievement of the goal should be independent of other (possibly related) goals	The course described in the syllabus does meet the “RCR training requirement”, but it also fulfills two useful functions (introducing a code of conduct and orienting students towards ethical reflection)	Requiring students to achieve the target level of performance does not require specific courses to be offered (so using this matrix cannot support a “compliance” approach to “RCR training”)
4. Goal should be actually and observably amenable to an active intervention	Syllabus describes levels of participation in detail—all are observable and assessable- by the instructor and by the students themselves	
5. Achievement of the goal should be documented/documentable with either quantitative or qualitative <as appropriate> outcomes	Final assignment represents the students’ self assessment and utilizes their semester’s worth of written work to justify their assessment (equally true for faculty assessment of each student)	
6. Achievement of the goal should result in a change that is detectible and meaningful	Students who participate in a course structured like this one will demonstrate skills (ethical reasoning) and knowledge (professional conduct) that are currently not observed; the change will be meaningful; engagement with ethics training for Big Data practitioners would be a complete change that would be meaningful	
7. Goal should be feasible	The course syllabus is complete—offering or adapting this course is feasible	

It can be seen that syllabi organized such as those given in Appendices 1 and 2 will meet the NAE ethical training goal criteria. A course organized using Appendix 2 will also meet NIH (2009) and NSF (2009) training requirements for “responsible conduct of research”. Finally, courses such as these also achieve the overarching goal of teaching “…people who want to do good science about the ethical standards and issues in their work, and how to deal with ethical problems that they encounter as scientists” (Swazey and Bird 1997: 5). As is outlined in the historical summaries, these codes of professional conduct were created from within the disciplines, and based on professional practitioner experiences “with ethical problems they encounter”.

## Discussion

The codes of professional conduct we have outlined clearly show that the decisions that go into the everyday *practice* of these disciplines, as well as others in STEM, are expected to be grounded in professionalism and responsibility. This is true whether or not practitioners support research specifically. The historical summaries describe the development of their respective codes, but also represent how each discipline assumes its membership will adhere to a code of professional ethics, with neither group formally inculcating its membership to this code.

To date, discussions of “ethics” in Big Data (research or commercial applications) tend to be focused on privacy issues relating to the collection—and not the use—of Big Data (e.g., Ringelheim 2007; European Union 2009; Federal Trade Commission 2012; White House 2012; Polonetsky and Tene 2013). For example, a 2009 edited volume, “The Fourth Paradigm: Data-Intensive Scientific Discovery” (Hey et al. 2009), has one chapter (of 28 content chapters) that focuses on policy (“The future of data policy”), with nothing specifically on the ethical use (or collection) of Big Data. In contrast, Dwork and Mulligan (2013) suggest that the focus on privacy and its protection are targeted by the computer science, policy, and software engineering domains because such solutions are easy (or, easier than the other, more complex problems), stating, “The ease with which policy and technical proposals revert to solutions focused on individual control over personal information reflects a failure to accurately conceptualize other concerns” (see Dwork and Mulligan 2013 for discussion of what other than privacy should be concerning Big Data as of September 2013). Our point is that the relevant ethical issues cannot be predicted, and they currently cannot even be agreed upon in some cases; however, the ability to reason ethically is a stable—learnable and improvable—skill set that students can begin to develop. Engaging a professional code of conduct to introduce ethical reasoning can bring the idea of professionalism, and possibly relevance, to training in ethics. We argue here that this is an important consideration for Big Data practitioners in training, but this is important for adequate preparation in *all* sciences.

Like computer experts in the late 1940s, Big Data researchers are an inchoate group of technical experts who (currently) only share a general set of tools and techniques in common. The ACM and ASA communities eventually coalesced around their respective common professional aspirations or associations; like statistics at its inception, Big Data draws its practitioners from across and within a variety of disciplines. However, unlike statistics and computing machinery, “Big Data” in the 2010s is a term that does not represent a coherent or stable community of practice or academic discipline, but rather a collection of tools used to collect and manipulate large data sets. Therefore, practitioners of Big Data are unlikely to arrive at a single, uniform code of professional conduct. We have yet to see within Big Data research or practice a concerted drive for professionalization, in the shape of an overarching membership organization, a distinct identity of a research community, an organized study of educational curricula, or a widely-shared code of ethics or professional conduct. However, we have also seen that two disciplines contributing to Big Data have all of these things except a “widely-shared code of ethics”—the codes are published, but they are not shared or taught.

The MR-ER is clearly supportive of, and aligned with, the sort of training that would promote the introduction of a code of conduct such as those outlined by the AoIR (Association of Internet Researchers 2012), ASA (e.g., see Tractenberg 2013), or ACM. It is also consistent with existing RCR training materials (e.g., Steneck 2007) and also promotes curriculum development and evaluation explicitly. The MR-ER is focused on decision-making and reasoning, two critical elements required for thoughtful contemplation of ethical, legal, and social issues (with respect to Big Data, or any field; e.g., Tractenberg et al. 2014; see also Schmaling and Blume 2009.).

There is currently no formal integration of the ASA code of professional conduct into master’s or doctoral training of statisticians (ASA Executive Director, personal communication, July 2013). We were similarly unable to find formal incorporations of the ACM and AoIR recommendations for ethical research into any of the graduate programs described in online materials in the U.S. This suggests that, although our emphasis here is on bringing some structure and support to the integration of ethical training into the preparation of individuals who will work in/with Big Data, our focus could be relevant to other disciplines as well. We propose that academic disciplines that train students to collect and/or use Big Data should incorporate formal training in ethical reasoning—irrespective of whether there are specific (e.g., federal funding) requirements for training in the “responsible conduct of research”. Although our approach is to introduce new practitioners to ethical reasoning and professional conduct with a single semester course, the MR-ER framework also promotes ongoing engagement with both throughout the curriculum and along the entire career trajectory (Tractenberg and FitzGerald 2012; see also Powell et al. 2007).

Big Data research and practice need not wait until there is a single or even a “most relevant” professional organization before trainees and practitioners are introduced to, and oriented towards, ethical reasoning skills that support professional conduct and ethical research. Moreover, the approach we have outlined can be adapted for the code of conduct in any discipline. Individuals in leadership positions may be especially obliged to pursue this ethical development; the fact that training and achievement should differ depending on the level of the professional practitioner is one of our most important contributions to the discussion of ethical training that is required for competent, ethical, professional practice in and around Big Data. With the approach we have outlined, individuals can document their higher-level achievement, qualifying themselves to be these leaders and introduce the new professionals to these ways of thinking and being.

## Appendix 1: Teaching Materials Using the Mastery Rubric for Ethical Reasoning with the ACM code of professional conduct: Semester Course Syllabus

- **Ethical Reasoning for Big Data Scientists**
- Meetings: Monday 9–12
- *Location TBD*
- **Instructors:**
- **Office Hours: By appointment**
- **Overview:** This 3-credit course (pass/fail) is designed around two key learning goals: (1) to initiate a career-long developmental path for the reasoning skills required to explain and justify decisions regarding ethical questions and dilemmas; and (2) to learn as much as possible about the **eight fundamental ethical considerations** identified by the Association for Computing Machinery (ACM) as representing the key elements of ethical and professional conduct in this domain. You will also be asked to consider the role of “professional and ethical conduct” in your own work and collaborations—and to consider the purpose of this training for you, your colleagues, and your mentees or trainees.
- **Prerequisite or Co-requisite requirements:** Most workplaces provide guidance and information about regulations that bear on the profession or on employment, but these are typically not sufficient to constitute adequate training to promote professional and ethical conduct in some or even many situations that you might encounter. These training opportunities should be completed, but should *not* be considered adequate to complete your training in professional and ethical conduct.
- **Required text:** [*any readings that are critical either to guide or structure discussion*] The course is structured around the General Moral Imperatives that are outlined as Association of Computing Machinery ethical professional practice, http://www.acm.org/about/code-of-ethics. Familiarity with the code and these imperatives will support discussion as well as development of the reasoning skills in which the course is designed to teach and offer practice.
- **Orientation: The course is structured around, and assessments are based on the Mastery Rubric for Ethical Reasoning (MR-ER),** Rochelle E. Tractenberg & Kevin T. FitzGerald (2012): A Mastery Rubric for the design and evaluation of an institutional curriculum in the responsible conduct of research, *Assessment & Evaluation in Higher Education*, 37(8): 1003–1021. doi:10.1080/02602938.2011.596923
- **The paper is available here: doi:** 10.1080/02602938.2011.596923.
- **Mechanics of the course:** this course will meet weekly for a 3 h discussion, where your active participation is required. All course materials will be emailed (or handed out as appropriate).
  **Table:** Course objectives and topics
  Session topics	Objectives
  **Meeting 1:** Introduction/Methods/Mechanics	Describe the course purposes and structure, and the case study method for teaching; introduce the Mastery Rubric and understand the structure of each week’s meetings, writings, and assessment. *Assignment 1 given*
  **Meeting 2:** What is an ethical, legal, or social issue (ELSI)? ***First writing assignment due***	Discuss the utility of the prerequisite knowledge and how/whether augmenting this with formal ethical reasoning steps can serve as a basis for adequate reasoning and case study discussions. *Assignment 2 given*
  **Meeting 3:** Contributions to society and human well-being	Identify and articulate obligations to protect fundamental human rights and respect diversity in all cultures. Describe “socially-responsible use” of the efforts of Big Data scientists (and of Big Data itself). Discuss how training supports (or fail to support) the recognition of ethical or moral dilemmas (KSA 1). *Assignment 3 given*
  **Meeting 3:** Avoiding harm to others	Discuss decision-making frameworks (KSA 2) such as utilitarianism and social justice, and their relationships to cases or situations where which Big Data does, can, or could harm others. Explore how using such frameworks can (or cannot) support the avoidance of harm to others, or even the delineation of what is to be considered a harm by the various stakeholders in a given situation. *Assignment 4 given*
  **Meeting 4:** Honesty and trustworthiness in professional conduct	Discuss how identification and evaluation of alternative actions (KSA 3) with respect to choosing and deciding on courses of action. *Assignment 5 given*
  **Meeting 5:** Fairness and non-discrimination	Discuss decision-making and the justifications for the identification, management and/or removal of conflicts of interest or responsibility. *Assignment 6 given*
  **Meeting 6:** Honoring property rights including copyrights and patents	Reflect on decision-making in ethical dilemmas and how this supports, or fails to support, mentorship. *Assignment 7 given*
  **Meeting 7:** Give proper credit for/respect intellectual property	Using the ethical reasoning KSAs as a decision-making framework to work through case studies on intellectual property issues. *Assignment 8 given.* **Students select one of the 7 reasoning skills to emphasize in assignments from now on**
  **Meeting 8:** Respect the privacy of others	Using the ethical reasoning KSAs as a decision-making framework to work through case studies on privacy and how Big Data can and can’t respect personal privacy. Consider ownership of the actual data in any Big Data application. *Assignment 9 given*
  **Meeting 9:** Honoring confidentiality	Using the decision-making framework in the MR-RCR to discuss confidentiality within and outside of work teams; consider how confidentiality and privacy interact/intersect in Big Data applications, and how confidentiality and intellectual property interact/intersect generally. *Assignment 9 given*
  **Meeting 10:** General moral imperatives in professional conduct	Discuss the interaction of personal values, professional requirements, and social good in applications or development relating to Big Data. *Assignment 10 given*
  **Meeting 11:** Responsible authorship and publication, and peer review; collaborative research including collaborations with industry	Discuss decision-making for authorship and publication, and for your peer review of others (and the overall decision to obtain peer review) and the justifications for such decisions. *Assignment 11 given*
  **Meeting 12:** The scientist as a responsible member of society, contemporary ethical issues in biomedical research, and the environmental and societal impacts of scientific research	Explore the “stewardship” model of the scientist with respect to scientific disciplines, societies of scientists, and society at large. Reflect on decision-making in ethical dilemmas and how this supports, or fails to support, stewardship. *Final Assignment discussed/given*
  **Meeting 13:** Reasoning and ethical and professional conduct. ***Final writing assignment*** ***drafts*** ***due***	Discussion of the course, the ethical reasoning KSAs and their development, and the sense that students have of what they have learned and whether/how they might continue to learn. Discuss other ethics training paradigms and opportunities
  **7–10** **days later: Final writing assignment due**	
- **Instructional Methods and Approaches:** The course is structured around ethical reasoning, a framework for decision-making that follows a series of steps outlined in the Mastery Rubric for Ethical Reasoning paper. In each class meeting we will refer to—and utilize- the knowledge, skills and abilities listed in the Mastery Rubric (see manuscript).
  Students will submit a written analysis (500 words) of the case assigned for class **at least 2** **h** ***before*** **that week’s class meeting**. We will discuss this case as a group during the first 30–45 min of each class meeting; your case analysis will be the initial basis for your contributions to the discussion of the case. Students will use their case analysis and the Mastery Rubric to structure their contributions to the discussion.
  Following this case discussion, the instructors will then introduce another case, with information specific to that week’s topic area. The next 90 min will then be focused on an in- class analysis and evaluation of the new case and supplemental information, again using the Mastery Rubric knowledge, skills and abilities. Cases will be selected from RCR training resources, and from current biomedical literature. Our purposes in analyzing and discussing the cases are to work through the ethical reasoning framework in the MR-ER and use these reasoning skills to explore and improve our understanding of the ACM General Moral Imperatives.
  The final part of the class time will focus on a summation and integration of what has been discussed, and an introduction to the topic and objective of the next class. After the class meeting, students will submit a revision of their initial case analysis that reflects the group discussion of the analyses submitted prior to class; this second analysis will incorporate additional points from the assigned readings or elements of the discussion that the individual student agrees or disagrees are important, and highlights one of the seven MR-ER reasoning skills that is the focus of that week’s work.
  The first eight meetings of the course are focused on orienting students to the knowledge, skills, and abilities in the Mastery Rubric for Ethical Reasoning, utilizing the ACM moral imperatives list as content on which to practice each of these. After the eighth meeting of the course, we will shift from exploring and initiating development in, and practice of, the knowledge, skills and abilities to refining individual confidence with each. So for the remaining meetings, this second analysis will be focused on whichever of the seven knowledge, skills, or abilities that the student believes is most salient for considering, or resolving, the case, or one of the KSAs that the student feels they need more practice with/feedback on.
  It is this second, revised submission—that includes a synthesis of facts with experience and discussion, plus reflective reasoning—that is graded.
- **Performance Evaluation:** Achievement is determined by assessment of the written case submissions (with scoring as outlined below), individual class discussion contributions, and the final assignment. Discussion contributions are opportunities for participants to improve their abilities to assess their own performance. The final assignment [elaborated below] is for each participant to selectively use their second analyses from all meetings to propose, and *support* their perception of, what overall level in the MR-ER they have achieved.
  We will introduce and review the final assignment in 12th meeting with the first draft turned in 2 h prior to the final class meeting, and final version turned in 10–14 days later. We will email you more information, including worksheets and table templates, to use in structuring your final assignment draft(s) and final version.
- **Assignments and Grading:** Maximum total points for the course is 100. Four points are possible for the second written analysis each of 10 weeks (max = 40); however, if the first case write up was not turned in on time, only three (of four) points possible for the second write up. Each essay will be evaluated according to a reflective writing rubric that will be emailed to participants. Formative feedback will be provided on the ungraded first analysis, in keeping with the Mastery Rubric, to support and strengthen the learner’s growing skill set.
  Your active participation in each class meeting will be worth 4 points (max = 40). The final class meeting and final essay will each be worth 10 points. Your final essay will be graded, not on the level the student believes s/he has attained, but on the *quality of the argument* supporting whatever level is identified. This is a course on reasoning, and the final project is a demonstration of your reasoning skills.
- **Final Assignment:** Now you have explicitly linked your knowledge and your prior experiences to KSAs, so that your 500 word essays (assignments) represent evidence of your having demonstrated each KSA to some level. Your final assignment is to use 1,000 words (if you need to) to describe—using your essays to support your reasoning as evidence of your current level and how it has changed over the semester—your current level of performance on each KSA. Unlike previous assignments, this one asks you to look at your performance of each KSA at the start of the class and now, and describe to us -using your own homework—whether and how your KSA performance has changed over the semester.
  Don’t just say, ‘I am at level X because I wrote 10 essays’. You want to say something like, ‘I am NOW a beginner on each KSA and I know I am at the beginner level because (A) here’s how that KSA has changed over the semester (using evidence from at least two essays from different points in the term); and (B) and here’s the evidence of how I know my functioning on this KSA is at the beginning level.
- Keep in mind: you’re not stating that you cannot or did not go beyond the Beginner level. We want you all to write similarly-*arranged* essays, so that without question, everyone who finishes this course can feel confident they’ve attained AT LEAST the beginner level. That doesn’t mean that, in one more week’s time, you couldn’t do this again and show/justify that you’re at the competent level. The assignment is simply to focus your attention at the beginner level, using your evidence—and your ability to think about your writing as evidence of your own reasoning. You are writing to a target, using the structure of the KSAs and MR-ER that are the focus of the course.
  Then, please take not more than 500 more words to describe what you learned during the course, and how did either the course overall or specifically what you feel you learned change your thinking over the semester? It is ok if nothing changed—but whether you argue it has or has not, evidence never goes amiss! This isn’t a question about how you liked or didn’t like the course, but you can certainly reason that (for example), if only more people had brought their dogs to class, you would have learned SO much MORE. IF you think there was something structural about the course that you felt challenged you unnecessarily, or something that somehow prevented you from reaching your full reasoning potential, it’s very important for us to both know that AND to know that your perspective is well reasoned.

## Appendix 2: Teaching Materials Using the Mastery Rubric for Ethical Reasoning with the ASA Ethical Guidelines for Professional Practice: Semester Course Syllabus

- **Ethical Reasoning for Big Data Scientists**
- Meetings: Monday 9–12
- *Location TBD*
- **Course Objectives and Topics**
- NOTES: *Italics* = MR-ER KSA (2 sessions on each). Underline = NIH topics. **Bold** = ASA Ethical Guidelines for Professional Practice topic.

1. Orientation meeting: introduction to the KSAs, case study approach, and portfolio assessment. Begin discussion of *prerequisite knowledge*, and its role in developing a sense of responsibility for the conduct of research and the conduct of/practice quantitative science.
2. Discuss the utility of *prerequisite knowledge* and how/whether augmenting this with formal ethical reasoning can serve as a basis for adequate reasoning and case study discussions. Is it ever OK not to use the **highest possible levels of competence, judgment, and diligence in the design and execution of an analysis**?
3. Definitions of unprofessional conduct, research misconduct, and policies for handling **misconduct** in the workplace and/or from funders’ perspectives. Whistle-blowing, what promotes and what prevents it, and policies around whistleblowing and whistle-blower protections. Discuss *recognition of ethical or moral dilemmas* in these contexts.
4. Discuss *recognition of ethical or moral dilemmas* in the confidentiality and privacy interact/intersect in data science and quantitative applications, and how **confidentiality and intellectual property** interact/intersect generally.
5. Identify and articulate obligations to **protect fundamental human rights and respect diversity in all cultures**. Describe “socially-responsible use” of the efforts of data scientists. Discuss *decision*-*making frameworks* and their application(s) in protecting fundamental rights, ensuring social responsibility, and respect for diversity.
6. Describe *decision*-*making frameworks* and their applications in cases involving the design of **ethical** **clinical or animal research** , participant recruitment, and the concept of “informed” consent.
7. *Identify and evaluate alternative actions* with respect to current developments in **animal research/models** (e.g., translational research; power/sample size; relevance of model to human disease, etc.).
8. Discuss **responsibilities to funders, clients and employers**: identifying and avoiding conflicts of interest—personal, professional, and financial—in collaborative work and/or research. *Identify and evaluate alternative actions* in the identification, management and/or removal of conflicts of interest.
9. Discuss the use and interpretation of data analysis within and outside of work teams (collaborative work); responsibilities for *making and justifying decisions* with **due consideration of the employer or funder and funding structure** in data management, sharing, and ownership.
10. Whether or not quantitative work will be published or shared, **what are our responsibilities to our professional community**? Discuss *making and justifying decisions* around ethical dilemmas arising from the professional community perspectives including **publication, testimony** , and **peer review**.
11. *Reflecting* on the environmental and societal impacts of quantitative sciences in scientific research (academic or lay consumers). Discussion of the ***decisionmaking processes*** **every data scientist engages in**, whether in research or applied settings; and sole and team science contexts.
12. *Reflecting* on the quantitative scientist as a responsible member of society, and larger impacts of ***decisions*** **made by the quantitative scientist** throughout design and execution of analyses and simulations, and reporting of results.
13. Final meeting: final project/paper (to assemble a portfolio with a 1,000 word essay outlining how each completed assignment represents the learner’s growth and development in each KSA, and how the evidence represents their achievement of the “beginner” level—or how it does not); plans for future/continued growth by students/learners.
